# Lessons from Norovirus Outbreak in Warsaw, Poland, December 2012

**DOI:** 10.1007/s12560-014-9166-0

**Published:** 2014-10-18

**Authors:** Sylwia Kamińska, Żaneta Kruszewska, Elżbieta Lejbrandt, Małgorzata Sadkowska-Todys

**Affiliations:** 1 Department of Epidemiology, National Institute of Public Health-National Institute of Hygiene, Warsaw, Poland; 2 European Programme for Intervention Epidemiology Training (EPIET), European Centre for Disease Prevention and Control (ECDC), Stockholm, Sweden; 3 Department of Epidemiology, Local Sanitary Station, Warsaw, Poland; 4 Department of Epidemiology, Regional Sanitary Station, Warsaw, Poland

**Keywords:** Outbreak investigation, Foodborne, Viral disease, Norovirus, Poland

## Abstract

Efficient foodborne outbreak investigations are important for identification of gaps in food safety and public health practice. This article reports on an investigation of a gastroenteritis outbreak linked to catering food following a Christmas reception at the National Institute of Public Health-National Institute of Hygiene (NIPH-NIH) in Warsaw in December 2012. Of 192 employees eating food at the catering event, 97 (50.5 %) developed symptoms. Persons eating dishes with recipes containing frozen carrots were five times more likely to develop gastrointestinal symptoms compared to those who did not eat carrots. Laboratory analysis identified norovirus in stool samples taken from symptomatic persons. Leftover food was not available for testing. The investigators did not collect stool specimens from food handlers and did not conduct trace backs for the suspected food ingredients. This investigation underlines the need for a revision of an existing procedures and importance of their complementation with detailed instructions for the local public health authorities for effective completion of foodborne outbreaks investigations in Poland.

## Introduction

Foodborne and waterborne viral infections are increasingly recognized as cause of illness in humans due to observed changes in food processing and consumption patterns that lead to the worldwide availability of high-risk food. In Europe, foodborne viruses are key emerging human pathogens with regard to the number of outbreaks and people affected (Koopmans et al. 2002; Koopmans and Duizer 2004; Ostrek et al. 2013). The majority of infections are characterized by self-limiting symptoms of vomiting and diarrhea, with sudden onset and short duration (de Wit et al. 2001; Glass et al. 2009). Widespread, even multi-country outbreaks are possible. However, some foodborne outbreaks caused by viruses remain under-recognized, partly because of a lack of validated method for routine detection of viruses in food (Koopmans et al. 2003).

In Poland, outbreaks of acute infectious gastroenteritis must be notified to the local health departments. This is regulated by the State Sanitary Inspection Act of 1985 and Act on Preventing and Controlling Infections and Infectious Diseases in Humans of 2008. The Epidemiology and Food Safety sections of the local public health authorities conduct epidemiological investigations and take control measures. They communicate outbreak notifications and reports to the provincial health departments, which forward these to the National Institute of Public Health-National Institute of Hygiene (NIPH-NIH). On request of the local health authorities, the NIPH-NIH assists in outbreak investigation.

On 21 December 2012, an occupational physician at NIPH-NIH alerted the public health authority in Warsaw, reporting that 46 people had gastroenteritis following a Christmas reception with catering service held 2 days earlier. Food from the catering event was suspected to be a potential vehicle of infection. An investigation was initiated on the same day to estimate the extent of the outbreak, identify the causative pathogen, the vehicle, and source of the infection. As the outbreak occured at the NIPH-NIH, the Department of Epidemiology of NIPH-NIH was mandated to assist in the investigation.

We described this outbreak to identify gaps and opportunities for improvement for future investigations of viral foodborne outbreaks in Poland.

## Methods

### Case Definition

We defined a case as an employee of NIPH-NIH who ate food items served at the Christmas reception on 19 December 2012 and subsequently developed diarrhea (≥3 stools in 24 h) or vomiting within 1 week.

### Descriptive Epidemiology

The catering firm provided a list of all foods, desserts, and beverages served during the reception. Since all attendees were employees of NIPH-NIH, we searched for cases using the Institue’s e-mail distribution lists. The questionnaire included close-ended questions on food/beverages items eaten during or after the Christmas reception, symptoms experienced, and other potential exposures within the previous week. We excluded persons who did not eat food at the catering event. We described the outbreak by time, place, and person.

### Analytical Epidemiology

We conducted a retrospective cohort study among the attendees of the Christmas reception. We compared the risk of illness among employees who had consumed food items and beverages, comparing this with the risk in employees who had not consumed. We calculated food/beverage-specific attack rates (ARs), relative risks (RRs), and 95 % confidence intervals (CIs). We modeled the association between exposures and illness using log-binomial regression. We included in the model variables with *p* value of less than 0.2. We calculated the population attributable fraction (PAF) for vehicles significantly associated with disease in the multivariable analysis. We used STATA 12 (Stata Corp.) for the statistical analysis.

### Laboratory Investigation

The NIPH-NIH occupational medicine physician collected stool specimens and rectal swabs from symptomatic employees who gave their consent. We sent specimens to the microbiological laboratory in provincial health department in Warsaw, which cultured them for *Salmonella* sp*., Shigella* sp., and *Yersinia* sp. Stool specimens were also tested for adenovirus, rotavirus, and norovirus.

### Environmental Investigation

Inspectors from the local health department controlled the kitchen of the catering firm. They only interviewed kitchen staff regarding to possible gastrointestinal symptoms. They also inspected the cleanliness of the catering kitchen, the proper functioning of kitchen appliances. They did not collect swabs from kitchen surfaces for microbiological testing.

No leftover food items from the reception were available for testing. Specimens from kitchen staff were not collected.

## Results

### Descriptive Epidemiology

**Fig. 1:**
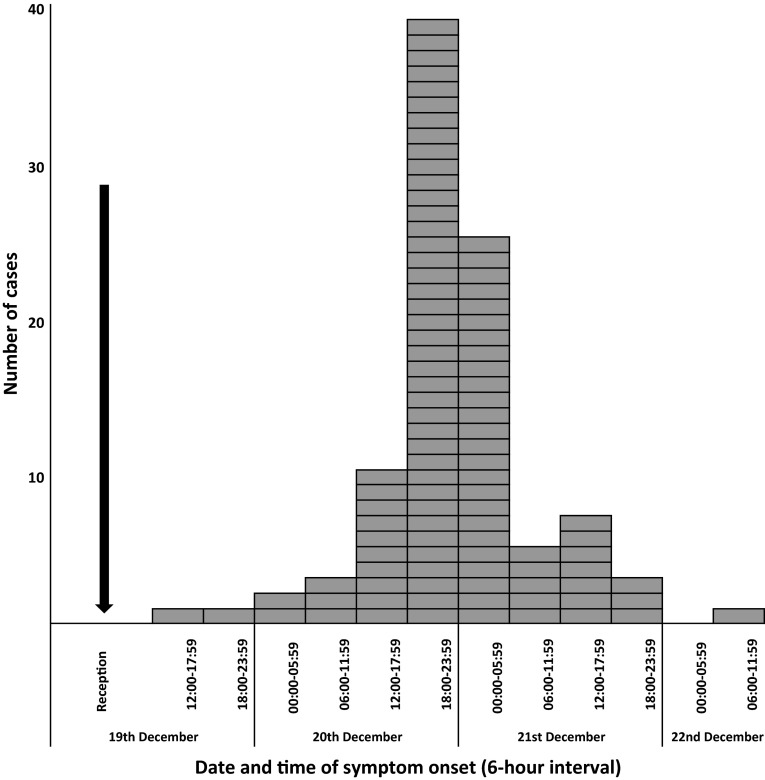
Cases of gastroenteritis by time of symptom onset, Warsaw, Poland, December 2012 (*n* = 97)

Of 306 employees contacted, 239 (78 %) completed the questionnaire. Of these, we included 192 employees who ate food from the caterer. Of these, 12 did not physically attend the reception but were offered dishes by colleagues who brought food to their offices. Of 192 persons at risk, 97 met the case definition. They were employees from all departments, 80 % were females, which reflects the gender distribution in the NIPH-NIH. The median age was 45 years (range: 25–69). Attack rate was 50.5 % (97/192); higher for female and any age groups less than 31 years and 51–60 (Table 1). Symptoms included abdominal pain (89 %), nausea (87 %), vomiting (78 %), diarrhea (69 %), and fever (56 %). None required hospitalization. All cases provided the time of their symptoms onset. Of these, seven reported symptoms within 24 h of the event (range: 6–72 h). The outbreak took place between 19 and 22 December 2012, with the majority of cases with onset of 20 December (Fig. 1). The pattern of the outbreak curve indicated a point source.

**Table 1:** Demographic details of persons who ate catering food during a Christmas reception, Warsaw, Poland, December 2012 (*n* = 97)

Demographic information	Cases	Total	AR (%)
Age (years)			
≤30	25	41	61
31–40	17	39	44
41–50	18	37	49
51–60	28	45	62
≥61	7	21	33
Unknown	2	9	22
Sex			
Female	78	148	53
Male	19	44	43

AR: attack rate

### Analytical Epidemiology

Consumption of eight food items was weakly associated with increased risk of infection (Table 2). Therefore, we hypothesized that many food items could be vehicles of this outbreak, either due to cross-contamination during food preparation/serving or using the same contaminated ingredients.

**Table 2:** Attack rate of gastroenteritis according to consumption of food items among consumers of catering food, Warsaw, Poland, December 2012

Type of meal	Exposure^a^	Consumed			Did not consume			Risk ratio			
								Crude		Adjusted^b^	
		Total	Cases	AR %	Total	Cases	AR %	RR	95 % CI	RR	95 % CI
Food items with frozen carrots	Vegetable salad	90	61	68	98	34	35	1.9	[1.4–2.6]	1.7	[1.2–2.2]
	Stewed fish with vegetables	67	48	72	122	48	39	1.8	[1.4–2.4]	1.5	[1.2–1.9]
	Carp in aspic	52	37	71	136	57	42	1.7	[1.3–2.2]	1.2	[1.1–1.2]
	Salmon cones with crab mousse	42	31	74	135	60	44	1.7	[1.3–2.2]	1.6	[1.3–1.9]
	Salmon rolls with vegetables	72	46	64	112	46	41	1.6	[1.2–2.1]	1.3	[0.9–1.7]
Food items without frozen carrots	Herring with sour cream	33	23	70	148	68	46	1.5	[1.1–2.0]	1.2	[0.9–1.6]
	Tatar steak	62	38	61	124	55	44	1.4	[1.0–1.8]	1.1	[0.8–1.6]
	Roasted meat (types of sausages)	77	46	60	112	49	44	1.4	[1.0–1.8]	1.3	[0.9–1.7]
Consumption of any food contained frozen carrots	Combined product	158	93	59	34	4	12	5.0	[2.0–13]	–	–

*AR* attack rate, *CI* confidence interval, *RR* risk ratio

^a^Food items and beverages provided by the catering firm

^b^Adjusted for carrots consumption

Multivariable analysis pointed to the consumption of vegetable salad (RR 1.7; 95 % CI 1.2–2.2), salmon cones with crab mousse (RR 1.6; 95 % CI 1.3–1.9), stewed fish with vegetables (RR 1.5; 95 % CI 1.2–1.9), and carp in aspic (RR 1.2; 95 % CI 1.1–1.2). All these contained frozen carrots as an ingredient.

Additional analysis indicated that eating any food item containing frozen carrots (6 food items) was associated with increased risk of illness (RR 5.00; 95 % CI 2.0–13.0) (Table 2). Seventy-seven percent of cases were attributable to eating foods containing frozen carrots (95 % CI 49.4–92.1 %).

### Laboratory Investigation

The occupational medicine physician collected four stool specimens and three rectal swabs. Of the four stool specimens, three were positive for norovirus; genotyping was not conducted. The laboratory did not identify other pathogens in the specimens.

### Environmental Investigation

The catering firm reported that all foods had been prepared on the day before the reception. The limited number of refrigerators did not allow for segregation of finished and semi-finished products. From the description of heat treatment process, we suppose that all food items prepared with frozen carrots were probably insufficiently processed. Inspectors found some products (i.e., flavorings for cakes, ready-to-use cake fillings) that their “used by” and “best before” dates have passed. However, those were not used for food preparation for this event. The owner of the catering firm was fined for identified negligence.

Food authorities did not trace back the frozen carrots identified as the most plausible vehicle of infection. They only reported that carrots were purchased frozen from one of the wholesale markets in Warsaw. The owner of the catering firm was not asked to present an invoice or any other document including information about the producer of the purchased carrots.

## Discussion

This gastroenteritis outbreak affected over half of the attendees of a Christmas reception at the NIPH-NIH, and was linked to contaminated catering food. Epidemiological investigations point to frozen carrots as the vehicle of infection. Results from the outbreak investigation identified a lack of key actions necessary for identifying sources of infections. Use of previously contaminated ingredients, improper food handling, or an infected employee who practiced poor personal hygiene at the workplace, could have contributed to the occurrence of this outbreak. However, lack of collection of specimens from kitchen staff, food leftovers, and the absence of attempts to trace back the frozen carrots made it impossible to identify the source of this outbreak.

The preliminary analysis pointed out the possibility of contamination of several foods. We identified the possible vehicle of infection by stratifying the analysis by food ingredients. Whether the food was contaminated before arriving at the catering firm or infection was due to poor food handling practices could not be determined since inspectors did not conduct a detailed environmental investigation.

Since food handlers were directly involved in food preparation, all catering firm’s kitchen staff should have be interviewed about food practices during the relevant periods (exact flow, handling, preparation, unusual circumstances, etc.), and knowledge about food safety practices. To exclude the possibility of contamination of the food during preparation by an asymptomatic carrier, it would have been essential to obtain stool specimens from food handlers (Parashar et al. 1998; Khuri-Bulos et al. 1994). If the same pathogen is identified both among food handlers and cases, it may indicate contamination during food preparation (Patterson et al. 1993; Maritschink et al. 2013). In many instances, asymptomatic carriers can contaminate food (Thornley et al. 2013; Nicolay et al. 2011; Ozawa et al. 2007).

Since the food inspectors were informed about the results of our analytical investigation, they should have considered a trace back of frozen carrots. Inspection of wholesale market and suspected batch producers could have helped verify the hypothesis that previously contaminated carrots were the source of infection. Lack of tracing did not allow implementation of any control measures to prevent continued contamination of the food chain. Since testing of food samples for enteric viruses is not routinely practiced in Europe (de Wit et al. 2001), and measures that can be taken to eliminate and reduce the virus in the food chain are limited, traceability of foodstuffs, and familiarity with the production chain in the food industry is increasingly important in ensuring the quality of ingredients. Thus, foodborne outbreak investigations could trigger early identification and efficient tracking of contaminations at different levels of the food chain.

Combination of trace back strategy and epidemiological analysis allows vehicle and source identification, or at least potential points of intersection in the manufacturing process of the food where contamination is most likely to occur (Isaacs et al. 2005; Buchholz et al. 2011; Vivancos et al. 2009). Effective product tracing could result in direct benefits, such as prevention of further outbreaks and sporadic infections. Effective product tracing can also result in improved public confidence and less disruption to commerce and markets. It may also provide benefit to firms (e.g., growers/breeders, food products sellers, and mass caterers) to help identify hot spots in a supply chain where food contamination can occur, and to implement better inventory control, improved supply chain management, and improved ability to meet regulatory requirements (Mejia et al. 2012; FERCO 2009).

We would like to emphasize the importance of performing a complete outbreak investigation, looking at all epidemiological, environmental and microbiological components, when viral outbreaks occur. In case of this outbreak, despite the causative agent of infection was known, the epidemiological investigation was inconclusive due to lack of food and food handlers testing.

### Limitations

We were not able to obtain a microbiological confirmation of the vehicle of this outbreak due to lack of testing food leftovers. Although foodborne viruses are increasingly studied, most laboratories involved in outbreak investigations in Poland (as well as in other European countries) do not have routine diagnostic methods for reliably detecting them in food. Nonetheless, in case of this outbreak if food samples were available, they would be tested for enteric bacteria, and positive result would be an evidence for fecal contamination. We were not able to verify the hypothesis about the food-handler as a potential carrier of norovirus due to the lack of stool specimens from kitchen workers.

## Conclusions

This investigation highlights that several gaps in the investigation of viral foodborne outbreaks in Poland still exist. These include particularly lack of routinely collected specimens from food handlers, not reviewing them in detail, and not performing trace back investigations of implicated products.

On the basis of our results, we suggest areas where changes could be of benefit. We recommend (1) revision of an existing procedures and their complementation with detailed instructions that need to be routinely followed when investigating foodborne outbreaks; (2) continuous training of all local inspectors which ensure rapid and exact identification of gaps in food handling to implement adequate corrective measures; (3) establish sensitive tests of detecting foodborne viruses in food samples.
